# Evidence of changing sexual behaviours and clinical attendance patterns, alongside increasing diagnoses of STIs in MSM and TPSM

**DOI:** 10.1136/sextrans-2020-054588

**Published:** 2021-08-19

**Authors:** Louis MacGregor, Nathan Speare, Jane Nicholls, Lindsey Harryman, Jeremy Horwood, Joanna May Kesten, Ava Lorenc, Patrick Horner, Natalie Lois Edelman, Peter Muir, Paul North, Mark Gompels, Katy M E Turner

**Affiliations:** 1 Department of Population Health Sciences, University of Bristol, Bristol, UK; 2 National Institute for Health Research, Health Protection Research Unit (NIHR HPRU) in Behavioural Science and Evaluation, Bristol, UK; 3 Bristol Sexual Health Centre, Bristol, UK; 4 Cardiff and Vale Sexual Health Service, Cardiff, UK; 5 National Institute for Health Research Applied Research Collaboration West (NIHR ARC West), University of Bristol, Bristol, UK; 6 School of Health Sciences, University of Brighton, Brighton, UK; 7 Public Health England South Region, Bristol, UK; 8 North Bristol NHS Trust, Westbury on Trym, Bristol, UK; 9 Bristol Veterinary School, University of Bristol, Bristol, UK

**Keywords:** syphilis, gonorrhoea, chlamydia infection, HIV pre-exposure prophylaxis, sexual behaviour

## Abstract

**Background:**

Due to rising numbers of STI diagnosis and increasing prevalence of antimicrobial resistance, we explored trends in STI testing frequency and diagnoses, alongside sexual decision making and attitudes concerning condom use and HIV pre-exposure prophylaxis (PrEP) at a large urban UK sexual health clinic.

**Methods:**

We examined 66 528 electronic patient records covering 40 321 attendees between 2016 and 2019, 3977 of whom were men who have sex with men or trans persons who have sex with men (MSM/TPSM). We also explored responses from MSM/TPSM attendees sent an electronic questionnaire between November 2018 and 2019 (n=1975) examining behaviours/attitudes towards PrEP. We measured trends in STI diagnoses and sexual behaviours including condomless anal intercourse (CAI), using linear and logistic regression analyses.

**Results:**

Tests resulting in gonorrhoea, chlamydia or syphilis diagnoses increased among MSM/TPSM from 13.5% to 18.5% between 2016 and 2019 (p<0.001). The average MSM/TPSM STI testing frequency increased from 1.5/person/year to 2.1/person/year (p=0.017). Gay MSM/TPSM had the highest proportions of attendances resulting in diagnoses, increasing from 15.1% to 19.6% between 2016 and 2019 (p<0.001) compared with bisexual/other MSM/TPSM increasing from 6.9% to 14.5% (p<0.001), alongside smaller but significant increases in non-MSM/TPSM from 5.9% to 7.7% (p<0.001).

The proportion of MSM/TPSM clinic attendees reporting CAI in the previous 3 months prior to at least one appointment in a given year increased significantly from 40.6% to 45.5% between 2016 and 2019 (p<0.0001) and average number of partners from 3.8 to 4.5 (p=0.002). Of 617 eligible questionnaire responses, 339/578 (58.7%) HIV-negative and 29/39 (74.4%) HIV-positive MSM/TPSM indicated they would be more likely to have CAI with someone on PrEP versus not on PrEP. 358/578 (61.9%) HIV-negative respondents said that PrEP use would make them more likely to have CAI with HIV-negative partners.

**Conclusion:**

Rising numbers of STI diagnoses among MSM/TPSM are not attributable to increased testing alone. Increased CAI and number of partners may be attributable to evolving sexual decision making among PrEP users and their partners. Proportionally, bisexual/other MSM/TPSM have the steepest increase in STI diagnoses.

## Introduction

In England, STIs diagnoses continue to increase with 450 000 reported in 2018, a 5% rise since 2017.^1^ Proportionally, rates of diagnosis of syphilis (*Treponema pallidum* (TP)), gonorrhoea (*Neisseria gonorrhoeae* (GC)) and chlamydia (*Chlamydia trachomatis* (CT)) are higher in gay, bisexual and other men who have sex with men (MSM) compared with heterosexual cisgender men and women and are highest among HIV-diagnosed MSM.^1^ Gonorrhoea cases increased the most (26% rise) with 47% of cases in MSM. Between 2009 and 2018, gonorrhoea increased 6.4-fold among MSM,^1^ which is especially concerning given rising antimicrobial resistance (AMR).^2–4^

The reasons for increased diagnoses of STIs in MSM are complex.^1 5^ These may include improved detection of STIs through increased testing, for example, more extra-genital testing in asymptomatic patients or usage of home/postal testing. Conversely, behavioural changes could increase STI transmission: increases in sexual partner numbers; condomless anal intercourse (CAI); group sex; and sexualised use of illicit substances to enhance or prolong sexual intercourse (chemsex) facilitated by geosocial networking applications.^6^ With more widespread knowledge of U=U and availability of pre-exposure prophylaxis (PrEP), decisions to move away from condom use as the primary prevention strategy among both people living with and without HIV alike may enable significant increases in bacterial STIs.

Since 2015, HIV incidence has declined due to increased HIV testing, treatment as prevention and increasing availability of HIV PrEP, an antiretroviral medication taken before and during periods of HIV risk, with high efficacy for preventing HIV acquisition.^7–9^ PrEP is currently available through National Health Service (NHS) sexual health services in Wales and Scotland^10^ and will be rolled out in England during 2020.^11^ Previously, PrEP has had limited availability in England, with free NHS access restricted to those enrolled on the Impact trial.^12^ In MSM and trans persons who have sex with men (TPSM), eligibility for the trial primarily involves reporting ongoing CAI or other factors posing similar HIV risk.^12^

There have been concerns that PrEP use could result in increased sexual risk-taking behaviours among HIV-negative MSM further increasing the incidence of STIs.^7^ Recent studies have given a consistent picture that the incidence of STIs in MSM increases following initiation of PrEP, but it is unclear whether this primarily reflects increased STI testing.^13 14^

We investigate factors contributing to rising STI incidence in MSM/TPSM by examining the trends from January 2016 to December 2019 in STI diagnoses, testing and sexual behaviours alongside attitudes towards PrEP use among MSM/TPSM attending a large non-London urban sexual health centre.

## Methods

### Population and setting

This work is part of the Challenges and Opportunities of PrEP (CHOP) Study, a mixed methods study combining analysis of: (1) routinely collected clinic data, January 2016–December 2019; (2) a self-completed online questionnaire carried out from 31 October 2018 to 31 October 2019 and (3) qualitative interviews with selected participants from (2). The study population are attendees of a large urban sexual health centre in Bristol, UK, which does not manage HIV-positive patients. For this paper, we consider clinic and online questionnaire data only; the qualitative interview data will be published elsewhere.^15^

### Electronic patient records (EPRs)

At each clinic visit, an EPR is completed, which contains all clinical information recorded during the consultation, tests undertaken and results. Each attendance is coded using the enhanced Genitourinary Medicine Clinical Activity Dataset (GUMCADv3), which has been used in Bristol since 2015. GUMCADv3 includes extended information on sexual behaviour including CAI, number of partners and recreational drug use, not recorded in GUMCADv2.^16^

We included all EPRs with a recorded STI test in our study period. Classification as MSM/TPSM was through self-identification as male gender (cis or transgender), with sexual orientation coded through the EPR as those who only have sex with men (gay) and those who have sex with men, women and/or other genders (bisexual/other). All attendees not meeting these criteria were classified as non-MSM/TPSM; this category included trans female attendees, due to the inability to reliably differentiate cis female and trans female attendees from the EPR dataset.

### Questionnaire

An electronic questionnaire was developed using Research Electronic Data Capture, including questions on sociodemographic characteristics, sexual health service use, sexual behaviour in previous 3 months, and views and experiences of PrEP. The electronic questionnaire link was distributed via SMS to all MSM/TPSM clinic attendees who consented to text communication with valid UK mobile numbers, for 12 months from 31 October 2018.

The inclusion criteria were: (1) consent to participate in the study; (2) indication that the respondent self-identified as MSM/TPSM (inclusive of both trans male and trans female respondents); and (3) complete answers on questions concerning condom use; HIV status (including ‘unsure’) and opinions and attitudes towards PrEP.

### Analysis

We describe the demographics of clinic attendees (EPR dataset) and questionnaire respondents, to ascertain representativeness of survey respondents. We compared age, ethnicity and index of multiple deprivation score categories using goodness of fit χ^2^ testing.

Using the clinical EPR data, we then examined the trends in STI diagnoses using linear regression, performing this analysis among both non-MSM/TPSM and MSM/TPSM attendees. We also evaluated trends in any CAI, number of partners and participation of chemsex (defined as use of crystal meth, mephedrone and/or GHB) in the 3 months prior to attendances alongside the proportion of attendances resulting in use of postexposure prophylaxis, following possible sexual exposure to HIV (PEPSE). For the purposes of this analysis, we define the number of ‘unique testers’ in a given year to be the number of individual MSM/TPSM attending for testing. We also define MSM/TPSM having only ever visited the clinic once for testing as ‘one-off testers’ and those having at least two STI tests within the clinic (at any time-point) as ‘repeat-testers’.

Finally, we examine CHOP questionnaire responses using univariate binary logistic regression models to calculate the crude ORs for the presence/absence of risk factors associated with STI acquisition. We define an independent categorial variable of: (1) PrEP users; (2) HIV negative or HIV status unsure (HIV negative/unsure) non-PrEP users; and (3) HIV-diagnosed MSM. Responses including missing data for variables of interest were excluded from the analysis.

We then describe questionnaire responses exploring how attitudes towards PrEP may influence changes in CAI. Specifically, we used an agreement scale (strongly disagree, disagree, not sure/don’t know, agree and strongly agree) in conjunction with the following statements: (1) ‘I would be more likely to have anal sex without a condom with someone I believe is HIV-negative if I was on PrEP’; (2) ‘I would be more likely to have anal sex without a condom with someone I believe is HIV-positive if I was on PrEP’; and (3) ‘I would be more likely to have anal sex without a condom with someone who was on PrEP than someone who was not on PrEP’.

All analyses were performed in STATA V.15.

## Results

### Demographics

Clinic records EPRs for 2016–2019 included 66 528 attendances (40 321 attendees) in which STI testing was undertaken. Of these, 10 794 attendance records (3977 attendees) were MSM/TPSM. Further breakdowns are provided in online supplemental figure S1. MSM/TPSM were primarily white 3429/3848 (90.7%), had sex with men only 3055/3975 (76.9%) and just over half were under 30 years 108/3975 (53.0%). Questionnaire invitations were sent to 1975 MSM/TPSM between 1 October 2018 and 1 October 2019, with 617 (31.2%) responses eligible for analysis. Questionnaire respondents were older, more ethnically diverse, but with similar indices of multiple deprivation compared with the clinical cohort (table 1).

10.1136/sextrans-2020-054588.supp1 Supplementary data

**Table 1:** Demographics of MSM/TPSM questionnaire participants and MSM/TPSM clinic attendees

Demographics	Number of questionnaire respondents	%	Number of clinic attendees*	%	N (df)† χ^2^ P value
Age (years)	**617**		**3975**		**4592** (**3**)
<30	198	32.1	2108	53.0	112.3
30–49	293	47.5	1467	36.9	<0.0001
>50	126	20.4	400	10.1	
Ethnicity	**617**		**3848**		**4465** (**7**)
White British	413	66.9	2906	75.5	37.7
White other	114	18.5	586	15.2	<0.0001
Mixed/multiple ethnic groups	29	4.7	89	2.3	
Asian/Asian British	34	5.5	142	3.7	
Black/African/Caribbean/black British	13	2.1	26	0.7	
Prefer not to say	9	1.5	71	1.8	
Other	5	0.8	28	0.7	
Highest qualification	**616**		–		–
No educational qualifications	10	1.6	–	–	–
GCSEs or equivalent	62	10.1	–	–	–
A-levels or equivalent	81	13.1	–	–	
BTEC/NVQ/diploma or equivalent	80	13.0	–	–	
University degree or higher	378	61.4	–	–	
Other	5	0.8	–	–	
Index of multiple deprivation (IMD)‡	**398**		**3759**		**4157** (**5**)
Quintile 1 (most deprived)	64	16.1	708	18.8	6.8
Quintile 2	89	22.4	860	22.9	0.1480
Quintile 3	99	24.9	751	20.0	
Quintile 4	72	18.1	767	20.4	
Quintile 5 (least deprived)	74	18.6	673	17.9	
Sexuality	**617**		**3975**		–
Sex with men	499	80.9	3055	76.9	–
Sex with men and women	64	10.4	918	23.1	–
Other sexual preference	54	8.8	2	0.1	
Gender	**617**		–		–
Cis male	599	97.1	–	–	–
Transgender	5	0.8	–	–	–
Non-binary/other	13	2.1	–	–	
HIV status	**617**		–		–
Unaware of their HIV status	26	4.2	–	–	–
HIV positive	39	6.3	–	–	–
HIV negative	552	89.5	–	–	
PrEP use (in HIV-negative MSM/TPSM)	**578**		–	–	–
PrEP eligible (total)	402	69.6	–	–	–
Has never used PrEP and is ineligible	155	26.8	–	–	–
Has never used PrEP but is eligible	221	38.2	–	–	
Is currently using PrEP and is ineligible	12	2.1	–	–	
Is currently using PrEP and is eligible	162	28.0	–	–	
Has previously used PrEP and is ineligible	9	1.6	–	–	
Has previously used PrEP and is eligible	19	3.3	–	–	

*Multiple visits may be associated with each clinic identification number; we use demographic information relating to the most recent visit between 1 December 2016 and 30 November 2019. Education, gender assigned at birth, PrEP use and HIV status could not be reliably discerned from clinical attendance records.

†χ^2^ test of goodness of fit, expressed with df and p value.

‡IMD is an overall measure of the relative deprivation based on geographical area of residence (specifically lower layer super output area).

BTEC, Business and Technology Education Council; GCSE, General Certficate of Secondary Education; MSM, men who have sex with men; NVQ, National Vocational Qualification; PrEP, pre-exposure prophylaxis; TPSM, trans persons who have sex with men.

### Testing trends

**Figure 1:**
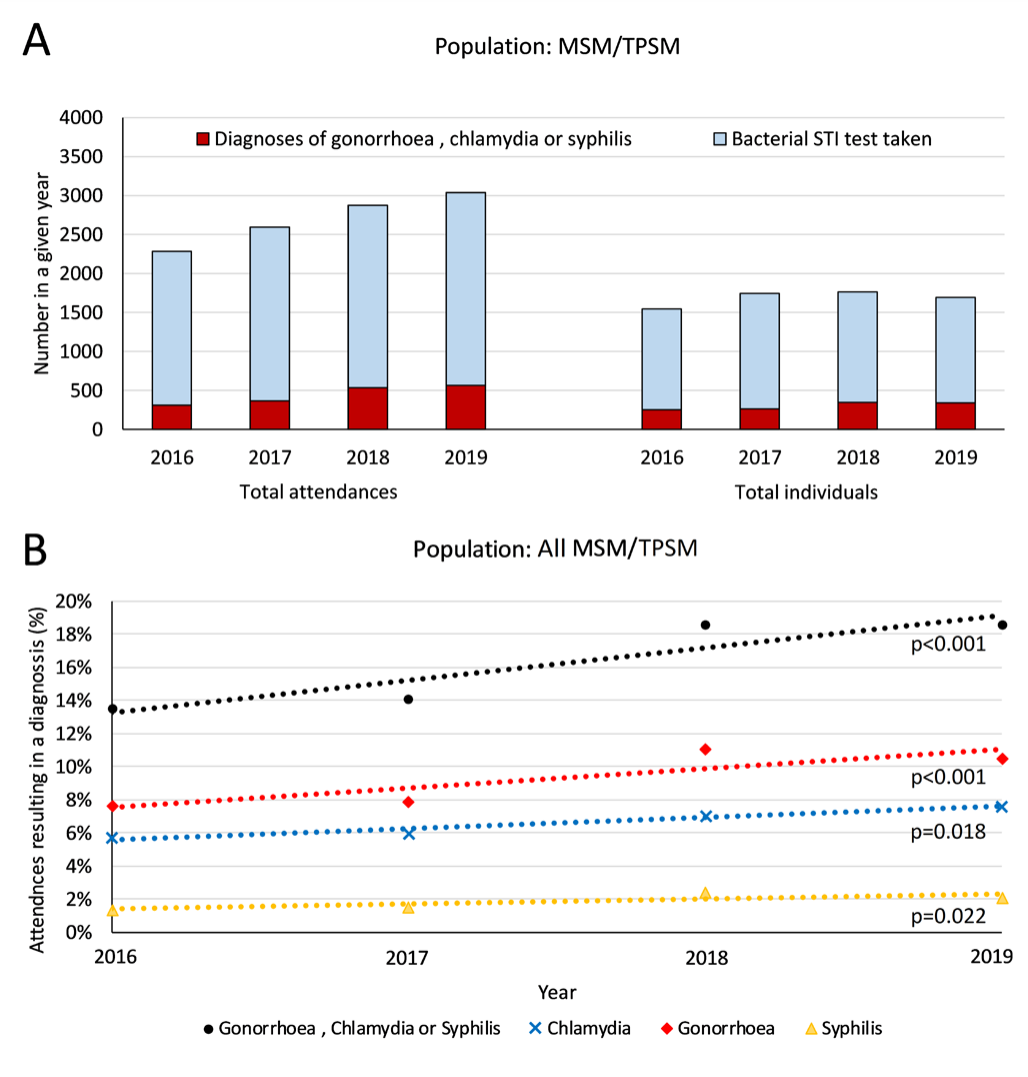
(A) Number of total MSM/TPSM attendances in which bacterial STI testing was undertaken between 2016 and 2019 and number of individuals attending clinic who had at least one bacterial STI test in a given year alongside the total number of associated diagnoses with STIs from these attendances or number of individuals attending clinic with at least one diagnosis of a bacterial STI in any given year. (B) Trends in chlamydia, gonorrhoea and syphilis diagnoses in MSM/TPSM among attendances in which bacterial STI testing was undertaken between 2016 and 2019, along with associated lines of best fit, using GUMCADv3 data from clinical attendances. MSM, men who have sex with men; TPSM, trans persons who have sex with men.

Clinical EPR data among MSM/TPSM indicated that the number of attendances at which an STI test was carried out increased from 2286 (2016) to 3039 (2019), an increase of 245 attendances per year (p=0.008) (figure 1). However, the number of unique testers (each year) remained stable (p=0.394), from 1545 to 1693. Regarding repeat-testers, tests taken per year increased on average from 1.5 to 2.1 (p=0.017).

**Figure 2:**
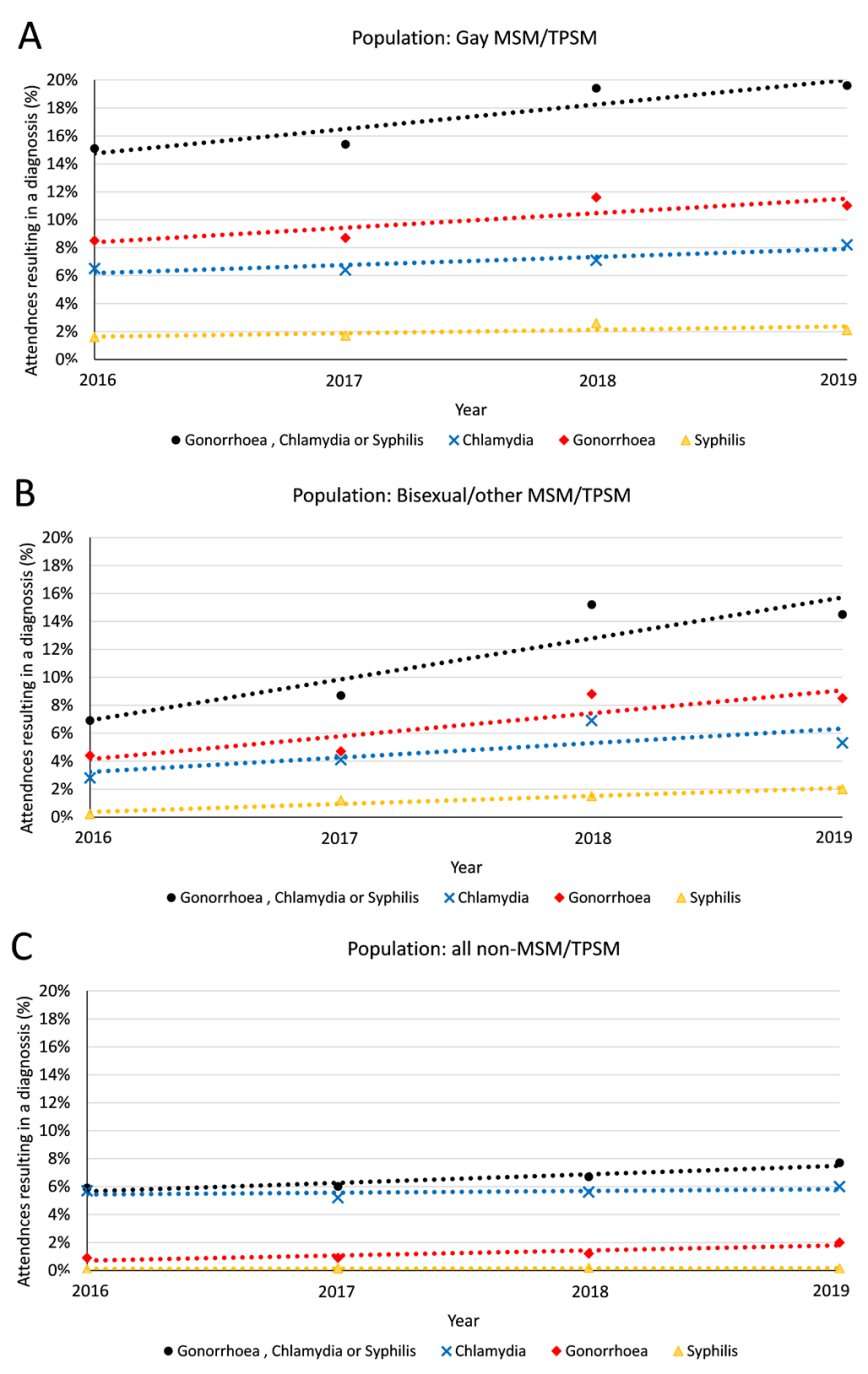
Trends in chlamydia, gonorrhoea and syphilis in (A) homosexual MSM/TPSM, (B) bisexual/other MSM/TPSM and (C) all other clinic attendees between 2016 and 2019, along with associated lines of best fit, using GUMCADv3 data from clinical attendances. MSM, men who have sex with men; TPSM, trans persons who have sex with men.

The clinical EPR data also indicated that among TPSM/MSM, the total number of bacterial STIs (CT/GC/TP) diagnosed increased from 309 in 2016 to 565 in 2019 and the proportion of attendances resulting in a bacterial STI diagnosis increased from 13.5% to 18.5% (p<0.001), with an annual increase of 94 infections per year (p=0.035). At least one of CT/GC/TP were diagnosed in 18.6% of individual attendees in 2016 compared with 27.4% in 2019 (3.4% increase per year; p<0.001). Gay MSM/TPSM had the highest proportions of attendances resulting in CT/GC/TP diagnoses in any given year (from 15.1% to 19.6%; p<0.001) compared with bisexual/other MSM/TPSM (6.9% to 14.5%; p<0.001), although the largest relative increase in the proportion of attendances resulting in diagnosis with CT/GC/TP was among bisexual/other MSM (figure 2). Similar trends were observed among non-MSM/TPSM clinic attendees; however, the proportion of attendances resulting in diagnoses were consistently lower than MSM/TPSM, increasing from 5.9% to 7.7% (p<0.001).

### Behavioural trends

The EPR data indicate that among MSM/TPSM, the proportion reporting CAI in the past 3 months during at least one appointment per calendar year rose from 40.6% to 45.5% between 2016 and 2019 (p<0.001), among repeat-testers increasing from 40.0% to 46.4% (p<0.001) but with no statistically significant change among one-off testers (p=0.065). The number of MSM/TPSM attendees who would be eligible for PrEP based on reported CAI increased from 627 to 771 (online supplemental figure S2), some of whom may already be using PrEP.

For MSM/TPSM attendees, the average number of partners reported (in the 3 months prior to the appointment) in all appointments in a calendar year increased from 3.8 to 4.5 (p=0.002), among repeat-testers increasing from 3.8 to 5.2 (p=0.001), again with no statistically significant change among one-off testers.

The proportion of MSM/TPSM attendees who had participated in chemsex in the past 3 months during at least one appointment in each calendar year rose from 2.1% to 3.3% (p=0.024), while the proportion prescribed PEPSE rose from 3.5% to 4.6% (p=0.033). The trends among repeat-testers and one-off testers for these behaviours were not significant; however, due to the rarity of these outcomes and the stratification of our population into subgroups, our analysis is likely to be underpowered in detecting these trends.

### Behaviours and attitudes towards PrEP

Among questionnaire respondents (of whom all were MSM/TPSM), 39/617 (6.3%) stated they were HIV-positive and 578/617 (93.7%) stated they were HIV negative/unsure, of which 174 (30.1%) were currently using PrEP. A total of 432 of 617 (70.0%) of respondents had engaged in CAI in the past 3 months. Furthermore, 42/615 (6.8%) had engaged in chemsex; 53/579 (9.2%) been prescribed PEPSE; and 42/582 (7.2%) had had rectal CT/GC or acute TP in the past 12 months, with breakdowns by HIV-status and PrEP use included in table 2.

**Table 2:** Associations between HIV risk factors and CHOP questionnaire respondent’s HIV status and current PrEP usage

Risk behaviours for HIV and demographic categories	Respondents in subgroup (%)	Number with risk factor (%)	OR* (95%CI)	P value
CAI in the past 3 months				
HIV negative/unsure and not on PrEP	404 (65.5)	240 (59.4)	1.0	
HIV negative/unsure and using PrEP	173 (28.2)	162 (93.1)	9.2 (5.0 to 17.1)	<0.001
HIV diagnosed	39 (6.3)	30 (76.9)	2.3 (1.1 to 4.9)	0.036
Diagnosis with a rectal gonorrhoea, chlamydia or acute syphilis in the past 12 months				
HIV negative/unsure and not on PrEP	375 (64.4)	19 (5.1)	1.0	
HIV negative/unsure and using PrEP	170 (29.2)	17 (10.0)	2.1 (1.1 to 4.1)	0.035
HIV diagnosed	37 (6.4)	6 (16.2)	3.6 (1.3 to 9.7)	0.011
PEP use in the past 12 months				
HIV negative/unsure and not on PrEP	404 (70.1)	28 (6.9)	1.0	
HIV negative/unsure and using PrEP	172 (29.9)	25 (14.5)	2.3 (1.3 to 4.0)	0.005
HIV diagnosed	- -	- -	- -	–
Chemsex in the past 12 months				
HIV negative/unsure and not on PrEP	403 (65.5)	9 (2.2)	1.0	
HIV negative/unsure and using PrEP	173 (28.2)	26 (15.0)	7.7 (3.5 to 16.9)	<0.001
HIV diagnosed	39 (6.3)	7 (18.0)	9.6 (3.3 to 27.4)	<0.001

CHOP, Challenges and Opportunities of PrEP; PEP, post-exposure prophylaxis; PrEP, pre-exposure prophylaxis.

The questionnaire data indicated that, compared with HIV-negative/unsure respondents not using PrEP, PrEP users were more likely to have had CAI in the past 3 months (OR 9.2 (95% CI 5.0 to 17.1, p<0.001)), participated in chemsex (OR 7.7 (95% CI 3.5 to 16.9, p<0.001)), been prescribed PEPSE (OR 2.3 (95% CI 1.3 to 4.0, p=0.005)) or been diagnosed with rectal CT/GC and/or acute TP (OR 2.1 (95% CI 1.1 to 4.1, p=0.035)) in the past 12 months.

Furthermore, a total of 339/578 (58.7%) HIV-negative/unsure questionnaire respondents and 29/39 (74.4%) of HIV-positive respondents stated that they would be more likely to have CAI with someone who was on PrEP than someone who was not on PrEP. While 358/578 (61.9%) and 162/578 (28.0%) of HIV-negative/unsure respondents said that if they were using PrEP, they would be more likely to have CAI with someone who they believed were HIV-negative/positive, respectively (online supplemental figure S3).

## Discussion

The number of attendances by MSM/TPSM for STI/HIV testing has increased by approximately 10% per annum between 2016 and 2019. This is as a result of more frequent testing per individual rather than an increase in unique MSM/TPSM attending the clinic. The total number of bacterial STIs (GC, CT and TP) diagnosed in MSM/TPSM also significantly increased, most notably in bisexual/other MSM/TPSM compared with gay MSM/TPSM. While bacterial STI diagnoses were less common for non-MSM/TPSM attendances, we also observed a significantly increasing trend. Behaviours associated with STI risk also increased among MSM/TPSM. With repeat-testers having the largest increases in frequency of CAI and average number of partners, potentially suggesting that health promotion messages regarding regular testing when at increased STI risk are working.

We find some evidence that rising incidence of bacterial STI diagnoses is due to an increase in partner numbers, CAI and chemsex drug use. Importantly, among survey respondents, PrEP users were more likely to report CAI and other risk factors for HIV and bacterial STI acquisition than HIV-negative/unsure MSM/TPSM not using PrEP. Interestingly, even among HIV-negative/unsure MSM, a potential partner using PrEP was frequently indicated to result in a higher chance of CAI, furthering evidence that community-wide increases in CAI could be driven by PrEP. This observation resonates worldwide, with rapid rises in PrEP uptake in Australia followed by rapid increases in CAI,^17^ CAI increasing in frequency among PrEP users in the Netherlands^18^ and rising PrEP uptake occurring alongside an increasing rate of CAI in the USA.^19^ Importantly, however, an increase in CAI was not an unexpected or even necessarily a negative consequence of PrEP.^20^ Given PrEP as an additional tool, MSM/TPSM are likely to consider and re-evaluate their own HIV risk management strategies^21 22^ against competing interests such as maximising sexual pleasure and feeling connected to their partners.^23^

The challenge however is to balance HIV risk and sexual pleasure, with the competing risks of STIs and the emergence and spread of AMR, particularly extensively drug-resistant GC, notably seen in the strains reported in recent UK case studies.^2–4 24^

A recent Australian study identified bisexual individuals as a possible bridging population between GC transmission clusters in MSM and heterosexual populations.^25^ This is of concern due to increasing bacterial STI diagnoses in both the MSM/TPSM and non-MSM/TPSM subpopulations, which are likely to be linked via mechanisms such as ‘bisexual bridging’. However, the direction and magnitude of this effect in our clinic population remains unknown and is beyond the scope of this study.

Another study defined the ‘PrEP-gap’ as the difference between MSM/TPSM currently using PrEP and MSM/TPSM who would be ‘very likely’ to use PrEP if they could access it.^26^ In the UK, PrEP use could triple from current rates to nearly 30% of the MSM/TPSM population. Given our findings and the current roll-out of PrEP across England,^11^ this could have significant consequences for condom use, continued increases in STI incidence and AMR.

The UK is not alone in experiencing rising rates of bacterial STI diagnoses, with rising trends being reported internationally.^27 28^ Understanding the influence of behavioural changes and the role of PrEP as an emergent part of HIV combination prevention is vital, with many countries committed to reducing the number of new HIV diagnoses and reaching the UNAIDS 90:90:90 targets.

The full interplay of increased testing, increasing PrEP use and the behavioural factors we have discussed such as CAI, increased sexual partner numbers and chemsex is difficult to determine and would benefit from further investigation, potentially using mathematical modelling. However, what is clear is that to tackle the issue of rising STI rates in the evolving landscape of PrEP, we must consider a holistic approach that addresses multiple factors, including facilitating MSM/TPSM to make informed decisions about the risks of CAI beyond HIV prevention strategies. We now face the challenge of addressing sexual health messaging in an era of reduced HIV anxiety and fear.^29^

### Strengths and limitations

This study brings recent and extensive data from a large urban setting outside London. Bristol is a Fast Track City^30^ committed to the elimination of new HIV diagnoses by 2030.^9^ Understanding the attitudes, STI trends and needs of cities outside of London remain a key endeavour to reach this target in the whole of the UK.

This study benefits from high data quality, resulting from use of the EPR mandatory fields (made uniquely possible in Bristol as a legacy of the GUMCAD3 pilot). We also used two complementary data sets, including a more detailed behavioural questionnaire to provide evidence to suggest how attitudes towards PrEP usage may lead to rising trends in CAI. Finally, this work takes a balanced approach in holistically examining multiple factors to explore the reasons for a continuing rise in common STI diagnoses in MSM/TPSM in the UK.

Due to convenience sampling and the subject material, the questionnaire respondents may be more likely to have an interest in PrEP or HIV prevention.^31^ Findings may not be generalisable to UK MSM/TPSM attending non-urban settings. Behavioural variables were self-reported and vulnerable to recall and social desirability bias. EPRs are clinician completed within the attendance and then updated to include processed results separately, potentially leading to missing information. Informal PrEP usage among clinic attendees was not available through GUMCAD coding, and knowledge of PrEP status was limited to responses given in the questionnaire. Furthermore, the EPR data did not include detailed information around gender identity, which meant we could not reliably determine all transgender attendees (in particular transgendered attendees identifying as female). This will be possible now that a revised GUMCADv3 has been implemented in all UK GUM clinics. The low number of transgender participants also meant that subpopulation analysis was not feasible. Lastly, from the EPRs, data were not possible to establish whether use of chemsex-related substances was solely during sexual intercourse.

In addition, the introduction of an online postal testing system in June 2017 may have affected in-clinic testing but is not link to other clinical attendee EPR records. The study clinic also introduced a rapid STI service for all symptomatic attendees in November 2018 with results of CT/GC available within 2 days. We however expect the impact of this new service on attendance patterns to be small, as this service was not advertised externally of the clinic and was only offered to attendees who were symptomatic and thus would have been likely to attend the service regardless of clinical pathway. Finally, people living with HIV were also likely to be testing for STIs at specialised services outside of the study clinic, and hence our sample of HIV-diagnosed MSM may not be largely representative of all HIV-diagnosed MSM.

## Conclusion

MSM/TPSM STI diagnosis rates are increasing, and higher testing frequency is a likely contributor, alongside changes in sexual behaviours and decision making. The highest proportion of visits resulting in STI diagnoses is among gay MSM/TPSM (19.6% in 2019), but the largest increase (6.9%–14.9%, 2016–2019) is among bisexual MSM/TPSM, which could lead to higher rates of STIs and AMR and thus increased morbidity and adverse health outcomes in other groups through bisexual bridging.

CAI and other risk behaviours are being reported more frequently across MSM/TPSM clinic attendees, especially among repeat-testers. MSM/TPSM taking PrEP, along with their partners (whether HIV negative or HIV positive), are more likely to have CAI compared with when neither partner is on PrEP.

Opportunities for public health and risk-reduction messaging for MSM/TPSM should be optimised for regular attendees. For example among PrEP users, to encourage informed decision making around CAI, which extends beyond anticipated increased risk of STIs to also include information regarding AMR and the potential of untreatable diseases. Public health understanding and awareness is likely to be facilitated by mass public experiential learning and education about infectious disease morbidity, transmission, spread and control measures as a result of the SARS-CoV-2 pandemic. Given the evidence of potential spread to non-MSM/TPSM populations and associated morbidity, MSM/TPSM should not be considered in isolation in terms of promoting regular testing, robust partner notification and sexual health promotion.

**Key messages**

- Among men/trans persons who have sex with men (MSM/TPSM), we observed increasing condomless anal intercourse, partner numbers and chemsex, alongside evolving sexual decision making in the era of pre-exposure prophylaxis.
- Among MSM/TPSM, the overall number of bacterial STI diagnoses are increasing, alongside an increasing proportion of attendances resulting in STI diagnoses and increasing testing frequency.
- The proportion of attendances resulting in bacterial STI diagnoses has increased across all populations including non-MSM attendees. The increase is most significant in bisexual MSM.
- Bisexual bridging could increase transmission between linked sexual networks, allowing STIs and antimicrobial resistance to move between different populations and impacting morbidity in other groups.

## Data Availability

No data are available. Due to the nature of this research, participants of this study did not agree for their data to be shared publicly, so supporting data are not available.
