# Supplementary material for: Evidence of changing sexual behaviours and clinical attendance patterns, alongside increasing diagnoses of STIs in MSM and TPSM
Source: Sex Transm Infect. 2021 Aug 19;97(7):507–13. doi: 10.1136/sextrans-2020-054588 (PMC8543230; doi:10.1136/sextrans-2020-054588)
Supplement: Supplementary data [file sextrans-2020-054588supp001.pdf]

Supplementary Material: Evidence of changing sexual behaviours and clinical attendance patterns, alongside increasing diagnoses of sexually transmitted infections in MSM and TPSM.

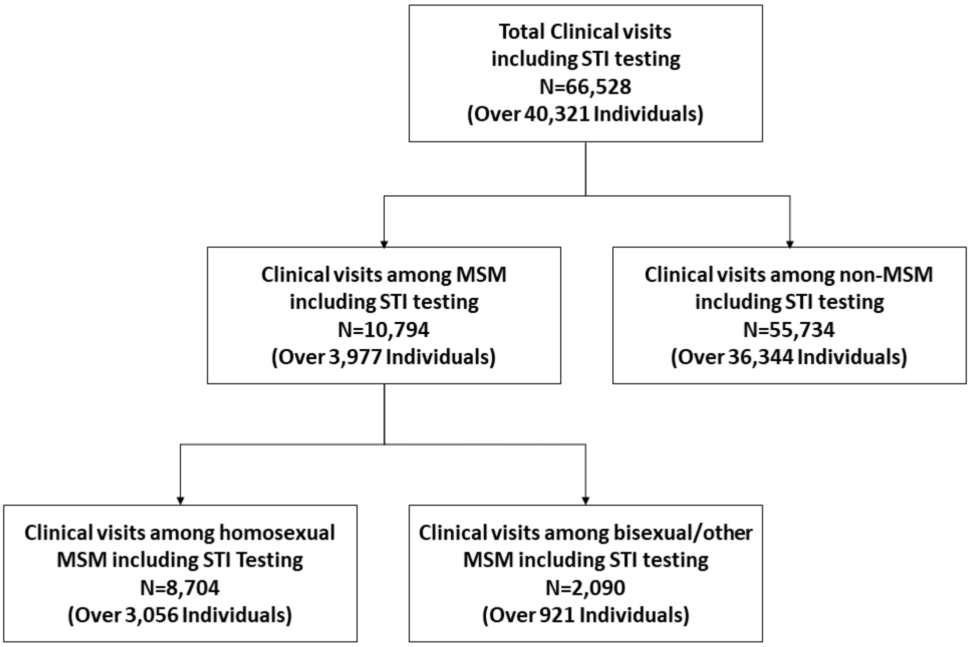

Figure S1: Flowchart of records by MSM/TPSM/non-MSM/TPSM and MSM/TPSM stratified by identification as gay MSM/TPSM or bisexual/other MSM/TPSM. Includes number of attendances and number of unique individuals.

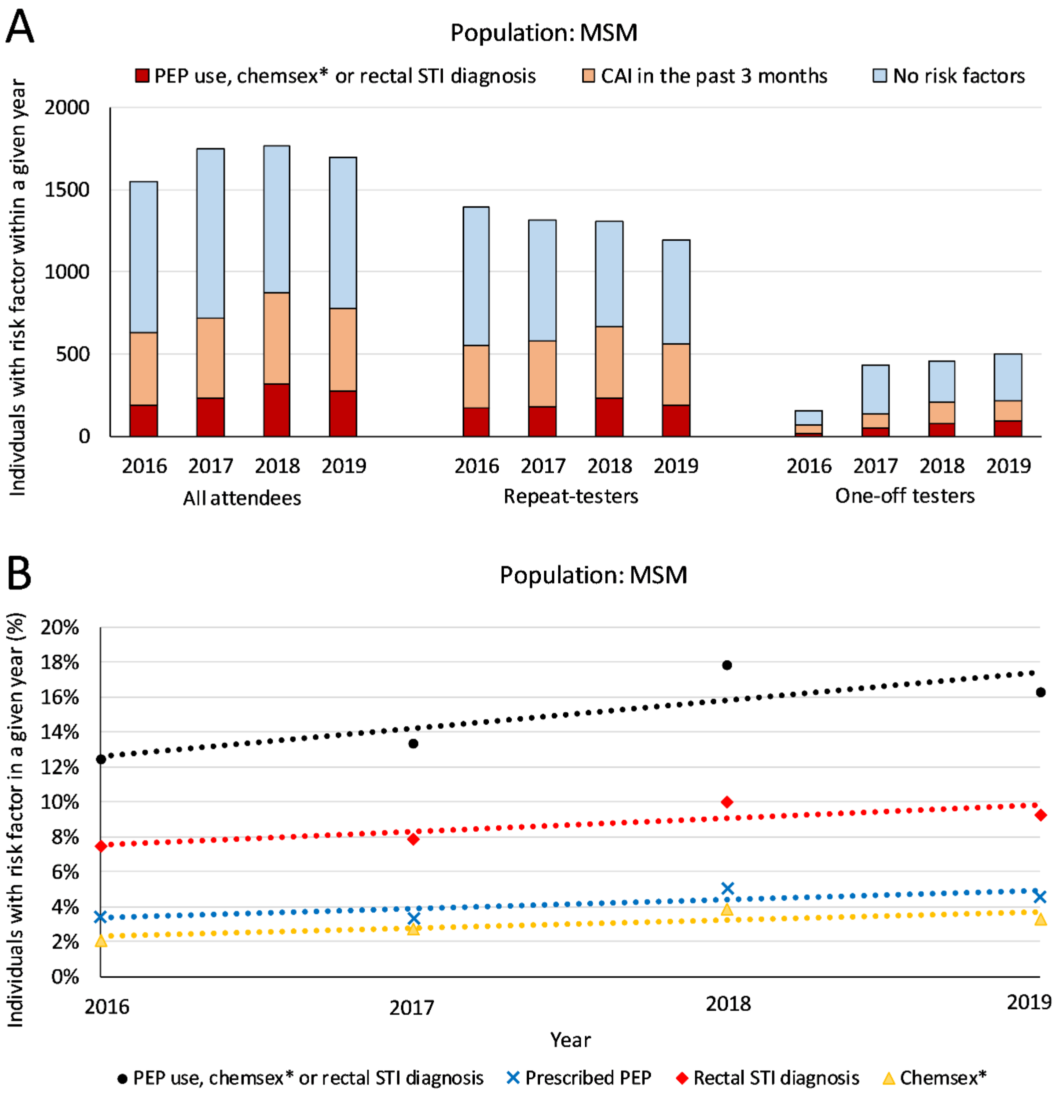

Figure S2. (A) Number of indivduals attending clinic who had at least one reported insance of CAI in the past 3 months in a given year alongside PEP use, diagnosis with a rectal STI or syphilis, or participaton in chemsex *3 months prior to clinic attendance test in a given year. Examined among all MSM/TPSM attendees, MSM/TPSM attendees who have only ever attended this clinic once (one-off testers), and those who have twice or more with at least one visit between 2016-2019 (repeat-testers). (B) Trends in CAI in the 3 months prior to clinic attendance alongside PEP use, diagnosis with rectal STI or syphilis, or participaton in chemsex *3 months prior to clinic attendance with associated lines of best fit, using GUMCADvs data from all MSM/TPSM.

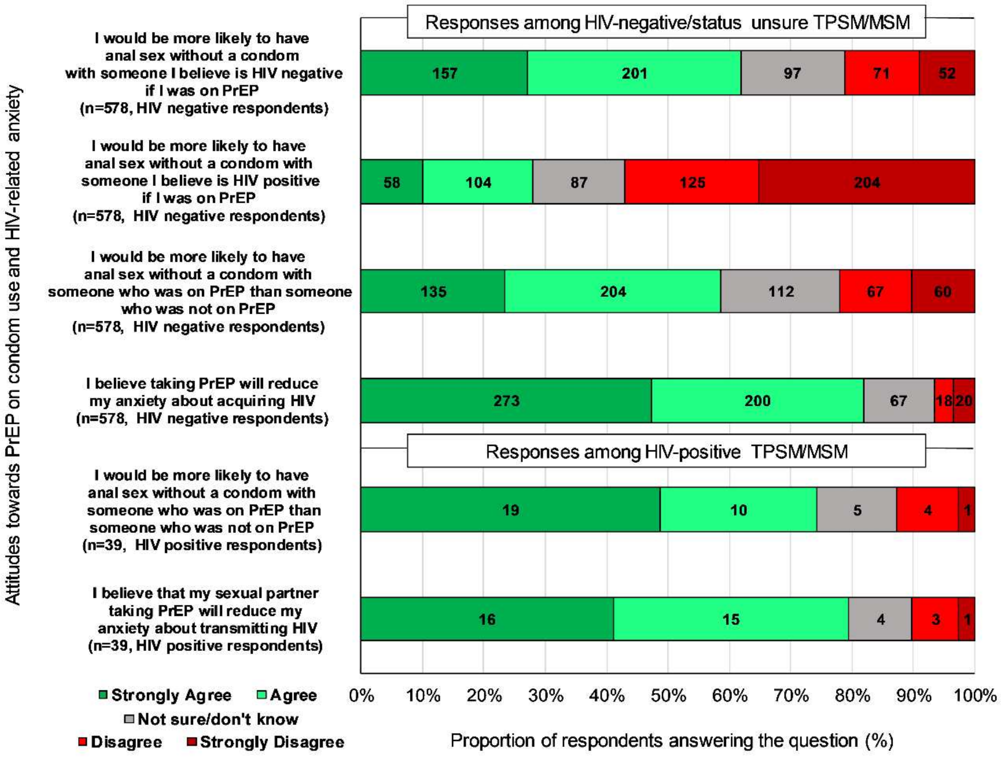

Figure S3. Proportion of TPSM/MSM/TPSM from the survey data who indicated agreement or non-agreement with statements concerning PrEP use and likelihood to use or forgo condoms.
